# Transcriptomic analysis reveals metabolic switches and surface remodeling as key processes for stage transition in *Trypanosoma cruzi*

**DOI:** 10.7717/peerj.3017

**Published:** 2017-03-08

**Authors:** Luisa Berná, Maria Laura Chiribao, Gonzalo Greif, Matias Rodriguez, Fernando Alvarez-Valin, Carlos Robello

**Affiliations:** 1 Unidad de Biología Molecular, Institut Pasteur de Montevideo, Montevideo, Uruguay; 2 Departamento de Bioquímica, Facultad de Medicina, Universidad de la República, Montevideo, Uruguay; 3 Sección Biomatemática, Unidad de Genómica Evolutiva, Facultad de Ciencias, Universidad de la República, Montevideo, Uruguay

**Keywords:** Chagas disease, Metabolic regulation, *Trypanosoma cruzi*, Surface proteins, RNA-seq

## Abstract

American trypanosomiasis is a chronic and endemic disease which affects millions of people. *Trypanosoma cruzi*, its causative agent, has a life cycle that involves complex morphological and functional transitions, as well as a variety of environmental conditions. This requires a tight regulation of gene expression, which is achieved mainly by post-transcriptional regulation. In this work we conducted an RNAseq analysis of the three major life cycle stages of *T. cruzi*: amastigotes, epimastigotes and trypomastigotes. This analysis allowed us to delineate specific transcriptomic profiling for each stage, and also to identify those biological processes of major relevance in each state. Stage specific expression profiling evidenced the plasticity of *T. cruzi* to adapt quickly to different conditions, with particular focus on membrane remodeling and metabolic shifts along the life cycle. Epimastigotes, which replicate in the gut of insect vectors, showed higher expression of genes related to energy metabolism, mainly Krebs cycle, respiratory chain and oxidative phosphorylation related genes, and anabolism related genes associated to nucleotide and steroid biosynthesis; also, a general down-regulation of surface glycoprotein coding genes was seen at this stage. Trypomastigotes, living extracellularly in the bloodstream of mammals, express a plethora of surface proteins and signaling genes involved in invasion and evasion of immune response. Amastigotes mostly express membrane transporters and genes involved in regulation of cell cycle, and also express a specific subset of surface glycoprotein coding genes. In addition, these results allowed us to improve the annotation of the Dm28c genome, identifying new ORFs and set the stage for construction of networks of co-expression, which can give clues about coded proteins of unknown functions.

## Introduction

*Trypanosoma cruzi* is the causative agent of Chagas disease, a chronic and endemic disease affecting millions of people mainly in America (http://www.who.int/mediacentre/factsheets/fs340/en/). This protozoan parasite has a complex life cycle involving both vertebrate and invertebrate hosts, and extracellular and intracellular stages (Brener, 1973). These environmental switches involve dramatic changes in the physiology of these parasites. In fact, *T. cruzi* has three main stages during its life cycle: trypomastigotes (infective and non-replicative), amastigotes (replicative and intracellular in the vertebrate host), and epimastigotes (replicative and insect-specific) (Brener, 1973; Vickerman & Preston, 1976).

These stages have been defined initially by morphological characteristics (Chagas, 1909) and, as expected, they imply changes at the cellular level, including surface composition and energy metabolism. Specifically, while epimastigotes are highly active in catabolism and anabolism related pathways, and potentially use nutrients from different origins (lipids, proteins, sugars) (Cazzulo, 1984; Cazzulo, 1992), trypomastigotes have low levels of transcription and translation, being specialized in attachment and infection of cells. In turn, amastigotes, although metabolically more active than trypomastigotes, do not have the versatility of epimastigotes in responding to different nutritional situations (Engel et al., 1987), even though very little information is available about amastigote metabolism.

Regarding the cellular surface, *T. cruzi* has a dense glycocalix formed by a large number of GPI-anchored proteins that to a certain extent constitutes an identity hallmark of these parasites (Acosta-Serrano et al., 2001). These surface proteins belong to several multigene families, product of gene expansion phenomena, which represents a characteristic feature of *T. cruzi.* Their biological relevance relies on the interaction with the immune system, resistance to low pH, and antibody clearance among others (Buscaglia, Campo & Frasch, 2006). These parasites are potentially able to remodel their surface, although large-scale studies of all the surface genes at the transcriptomic level were not performed up to date.

The above mentioned changes obviously require a fine regulation of gene expression. However, unlike most eukaryotes, trypanosomes have peculiarities in the genome organization and transcription. The genome of trypanosomatids is organized in clusters of protein-coding genes located on the same DNA strand, separated by relatively short intergenic regions (Daniels, Gull & Wickstead, 2010). With a few exceptions, genes do not contain introns, and the clusters are transcribed as long nuclear polycistronic units. This particular organization probably explains why only a few promoters have been found in trypanosomes. mRNAs maturation in trypanosomes involves *trans*-splicing and polyadenylation. *Trans* splicing is responsible for the addition of a capped spliced leader sequence (SL RNA) in the 5′UTR of each gene. Its mechanism is similar to that of *cis*-splicing. This process is coupled to the polyadenylation of the 3′ end of the gene located upstream on the same polycistronic RNA molecule. As a consequence, a molecule of mature mRNA (capped, polyA^+^, transpliced) is released from the polycistron and exported to the cytoplasm, where it can be translated. Therefore, in trypanosomes the 5′UTR is the sequence segment located between the SL and the first translated codon, whereas the 3′UTR is defined in the same way as in other eukaryotes. In contrast to bacterial operons, trypanosomatid polycistronic units do not contain genes that are functionally related. Moreover, despite their contiguity in the primary transcript, individual genes from the same transcription unit can show markedly different expression patterns (Vanhamme & Pays, 1995). This observation indicates that in trypanosomes regulation of gene expression operates mainly at the post-transcriptional level. The final outcome of protein production of trypanosomatids is indeed regulated at different levels with complex mechanisms. Recently, it has been demonstrated by ribosome profiling the relevance of mRNA translation efficiency in the abundance of specific proteins in *Trypanosoma cruzi* (Smircich et al., 2015) and other trypanosomes (Jensen et al., 2014; Parsons et al., 2015; Vasquez et al., 2014). However, in trypanosomatids only about 10 percent of the reads obtained by this technique are mappable due to the shortness of the sequence covered by the ribosome and the high amount of repetitive sequences and multigene families in these species. Therefore, some precaution is needed with the interpretation of the overall results. Regulation of gene expression in trypanosomes operates mainly at the post-transcriptional level, and numerous studies have demonstrated that 3′UTR regions affect mRNA stability, and hence differential expression (Clayton, 2016; Kramer & Carrington, 2011). Although the exact mechanisms allowing specificity are still unknown, some evidence has indicated that different domains in the 3′UTRs could explain, at least in part, changes in expression (Da Silva, Bartholomeu & Teixeira, 2006; Di Noia et al., 2000; Jager, Muia & Campetella, 2008). In spite of the importance of post-transcriptional changes, standard RNAseq analysis has proved to be a very informative tool for assessing expression profiles in trypanosomatids (Dillon et al., 2015; Fernandes et al., 2016; Greif et al., 2013; Kolev et al., 2010; Li et al., 2016; Siegel et al., 2010).

In this work we aimed to know which were the main transcriptomic changes during the life cycle of *T. cruzi*, with special emphasis on surface and energy metabolism remodeling. RNAseq of the three main stages of the parasite was performed, and allowed us to identify genes with important variation in their expression patterns (statically significant and with large size effects) at the RNA level. A systemic view about the features of gene reprogramming along the life cycle of *Trypanosoma cruzi* can be significant for future identification of key molecules to be used in the control of Chagas disease.

## Material & Methods

### Parasites

Epimastigotes were grown in liver infusion tryptose medium (LIT) supplemented with 10% heat inactivated fetal bovine serum (FBS) at 28°C (Robello et al., 1997). Trypomastigotes were collected from supernatants of infected monolayers of Vero cells (ATCC^®^CCL-81) in DMEM medium at 37°C under 5% CO2. Extracellular amastigotes were obtained by incubating trypomastigotes recently released from the cells in DMEM medium at 37°C under 5% CO_2_ for 24 h as previously described (Chiribao et al., 2012).

### RNA purification and quality control

Parasites were washed three times with PBS, and pellets directly lysed with Tri Reagent (Sigma-Aldrich, St. Louis, MO, USA). In order to obtain high quality samples the extracted RNA was further purified with IllustraRNAspin Mini Kit (GE Healthcare). Quantification was performed in a Qubit (Invitrogen), exhibiting a high content of total RNA, and quality was tested in a BioAnalyzer 2100 (Agilent Technologies), obtaining RNA integrity number (RIN) values above 8 in all the samples.

### RNA-seq library construction, quality control and sequencing

Directional libraries were constructed for each *T. cruzi* stage, by using oligo dT primers and reverse transcription. Quality control of the length of the library was done with BioAnalyzer DNA 1000 kit (Agilent Technologies), and quantification was performed with KAPA Library Quantification Kit (Kapa Biosystems). 15 pM of the libraries (mean length  = 350 nt), were clustered on an Illumina Single Read Flow Cell in cBot (Illumina). Single read 72 and 36 cycles of sequencing was performed on GAIIx instrument with Illumina Sequencing kits (TruSeq SBS v5-GA kit; Illumina). Raw data were deposited in the NCBI database under SRA accession number SRP072022.

### Bioinformatics and data analysis

For each stage, two libraries were generated of 36 and 72 bp each. Reads were filtered for ribosomal RNA, and a minimum of quality phred score of 20. After filtering a total of 43.98 × 10^6^, 40.52 × 10^6^ and 44.28 × 10^6^ reads of amastigotes, trypomastigotes and epimastigotes were obtained, respectively. Reads were aligned to the reference genome of *T. cruzi* Dm28c (24, 30-Mar-2015) using Bowtie (allowing two/three base pair mismatches for 36/72bp reads respectively).

To estimate transcript levels, we used ERANGE software that considers the unique regions of the genes to re-normalize the assignment of multimatching reads. The CDS plus an extension of 200bp at both sides (to the normalization process) were processed. The raw counts are presented in Table S9.

Differential expression analyses were performed using the R/Bioconductor package DESeq2.

Normalized counts were obtained from DESeq2 with the function count (dds, normalized  = T), and are presented in Table S9. Genes were considered as deferentially expressed (DEGs) when the following conditions were met: they were statistically significant as indicated by a FDR value lower than 0.05 (FDR is the False Discovery Rate, a correction of the *p*-value to account for multiple simultaneous tests) and had a fold change in transcript abundance of at least two (in either direction).

Gene Ontology enrichment analyses were performed using Tritrypdb tools (http://tritrypdb.org) with Fisher exact test filtering for false discovery rate (FDR) lower than 0.05.

Visual inspection of the alignment was performed using The Integrative Genomics Viewer (IGV) (Robinson et al., 2011).

In order to find possible new transcripts, libraries were pooled and aligned to reference genome using Bowtie (seed length 15, maximum mismatches in seed 1). The de novo RNA-Seq transcript assembly was performed using Cufflinks including the bias correction, the ‘rescue method’ for multi-reads and the 3′ overhang-tolerance set to 200 (Trapnell et al., 2010). TransDecoder was used to identify candidate coding regions within transcript sequences and open reading features (ORFs) smaller than 300 pb were discarded. Functional annotation of the translated ORF was done by HMM search (HMMER 3.1 (Mistry et al., 2013) against pfam database (Finn et al., 2014) and by Blastp search against nr NCBI (all non-redundant GenBank CDS translations) filtering for e-value lower than 1 × 10^−5^. Further filter including minimum alignment identity of 60%, minimum query alignment length 60% and, minimum subject alignment length 60% were incorporated to select complete genes.

3′UTR sequences of Dm28c genes were obtained from http://tritrypdb.org/tritrypdb/, extracting 400 pb after the transcriptional stop codon.

Alignments were performed with Clustalw2 (Larkin, Blackshields et al. 2007) and phylogenetic analysis with PhyML3.1 (Guindon et al., 2010). Modelgenerator (Keane et al., 2006) was used to select adequate substitution models. JTT was used for protein sequences and HKY85 was selected for nucleotide sequences. Visualization of phylogenies was performed with Figtree 1.4.2 (http://tree.bio.ed.ac.uk/software/figtree/).

For GPI identification we used PredGPI predictor (Pierleoni, Martelli & Casadio, 2008). A total of 782 proteins were identified to present at least one potential GPI-modification site.

## Results and Discussion

In the present work we have determined and compared the transcriptome profiling of the three main life cycle stages of the parasite *Trypanosoma cruzi*. PolyA^+^ RNA from parasites was purified, and libraries were constructed for amastigotes (A3 and A7), trypomastigotes (T3 and T7) and epimastigotes (E3 and E7), and sequenced by Illumina technology; after filtering for poor quality sequencing scores, we got around 4 × 10^7^ reads for each stage (Table 1). Reads were aligned to the reference genome of *T. cruzi* Dm28c using Bowtie (Langmead et al., 2009), allowing two mismatches for 36 nt reads and three mismatches for 72 nt reads. The amounts of reads that were of good quality and also aligned to reference genome for each library are presented in Table 1. As shown in this table around 60% of the reads map into the reference genome, which represents a significant proportion taking into account the still incomplete state of the Dm28c genome assembly.

**Table 1:** RNA-seq mapping statistics.

Library	Read length	Total reads	Aligned reads	% Aligned reads	Read counts in CDS^*^	% read counts in CDS^*^
A3	36	37.972.908	21.083.527	55,5	10.150.097	48,1
A7	72	6.011.870	2.644.677	44,0	1.314.640	49,7
T3	36	37.549.496	24.880.138	66,3	10.941.002	44,0
T7	72	2.976.919	1.604.848	53,9	716.431	44,6
E3	36	35.740.671	24.024.322	67,2	9.482.211	39,5
E7	72	8.540.435	5.496.063	64,4	2.192.983	39,9

**Notes.**

* CDS extended 200bp at both sides.

A amastigotes T tryomastigotes E epimastigotes

In order to determine and quantify the transcript levels of each gene in the different stages, reads were assigned to coding sequences (CDS) using Enhanced Read Analysis of Gene Expression ERANGE (Mortazavi et al., 2008). Basically ERANGE assigns reads that map uniquely in the genome. For those reads matching equally to two or more sites, this program uses the extended coding sequences (we have chosen 200 nt at both sides of the CDS, see Methods) assigning them to their most likely site. Total reads counts are presented in Table 1. It is noteworthy that 50% of the mapped reads do not map in CDS but in other regions of the genome (Table 1). Visual inspection of the density (sequencing depth) of mapped reads suggests that several reads mapped on UTRs regions, predominantly on 3′UTR. It should be mentioned that *T. cruzi* UTR lengths vary according to the gene size, and have been estimated from experimentally mapped genes to range from 10-400 bp for 5′UTR and 17-2800 bp for 3′UTR (Brandao & Jiang, 2009), being 3′UTR 2-3 times longer than its corresponding 5′UTR (Ziccardi & Brandao, 2011). This result reinforces previous findings showing the relevance of 3′UTRs in the regulation of gene expression (Coughlin et al., 2000; Da Silva, Bartholomeu & Teixeira, 2006; Di Noia et al., 2000; Nozaki & Cross, 1995; Weston, La Flamme & Van Voorhis, 1999).

A second point that needs to be considered here is that most of the sequenced *T. cruzi* genomes are still in a “draft-like” form, mainly due to the high number of repetitive sequences. Indeed, *T. cruzi* genome consists of more than 50% repeats that include surface molecule genes and several other gene families, as well as the poorly characterized retroelements (Arner et al., 2007; El-Sayed et al., 2005). Additionally, we cannot discard that many assembled regions are not completely annotated. In this regard the RNA-seq data can be used to help detect novel transcripts and new genes. In the following sections we will focus our analysis on surface genes, metabolic pathways and the annotation of potentially new genes.

### Highest expressed genes

Analysis of 500 most expressed genes revealed that 277 genes are common to epimastigotes, amastigotes and trypomastigotes. Gene ontology enrichment showed that these genes are related to microtubule movement, chromosome organization, DNA packaging and conformation change, response to stress, cell cycle progress, chromatin assembly among others (Table 2 and Table S1). These results suggest the relevance of epigenetic regulation in *T. cruzi* life cycle. Also amino acid activation (synthesis of aminoacyl-tRNAs) and other proteins related to translation machinery appear to be relevant in driving changes through the cycle (Table S1). Metabolic pathway analysis of shared highly expressed genes showed that aminoacyl-tRNA biosynthesis, purine metabolism, glycolysis and porphyrin metabolism are the most important. Protein synthesis, folding and degradation pathways were also very represented in this group with many ubiquitin-proteasome system genes, translation factors and chaperones being greatly expressed. It is noteworthy that some aminoacyl-tRNA synthetases show high expression profiles: glutamyl, isoleucyl, prolyl and valyl-tRNA synthetases. We cannot discard that these enzymes might have additional domains and roles (Table S7). It is known that leucyl-tRNA synthetase participates as a sensor that mediates amino acid dependent mTORC1 activation (Han et al., 2012) and glutaminyl-tRNA synthetase participates in the antiapoptotic activity of glutamine by its interaction with ASK1 (Ko et al., 2001). Due to the relevance of proline in parasite differentiation (Contreras et al., 1985; Tonelli et al., 2004) it is tempting to speculate that prolyl-tRNA synthetase might also be involved in sensing and/or regulation roles. Activation of translation, expression of aminoacyl-tRNA synthetases, folding and ubiquitin proteasome expression suggest that besides epigenetic control, protein remodeling also plays a relevant role in parasite stage transition.

**Table 2:** Gene Ontology enrichment of commonly highest expressed genes.

GO term	% genes present	Fold enrichment	*p*-value
Microtubule-based movement	21.0	5.4	2.73e−6
Cellular component movement	20.3	5.2	3.72e−6
Microtubule-based process	18.3	4.7	1.01e−5
Chromosome organization	35.3	9.1	1.46e−4
DNA conformation change	31.3	8.0	8.54e−4
Organelle organization	19.4	5.0	9.16e−4
DNA packaging	44.4	11.4	1.03e−3
Cellular component organization	12.3	3.2	5.39e−3
Cellular component organization at cellular level	12.7	3.27	7.75e−3
Cellular component organization or biogenesis	10.6	2.7	7.86e−3
Chromosome condensation	100.	25.7	7.94e−3
Sister chromatid cohesion	100.	25.7	7.94e−3
Chromosome segregation	100.	25.7	7.94e−3
Response to stress	12.1	3.1	9.99e−3

### Differential expression of *T. cruzi* surface genes

**Figure 1:**
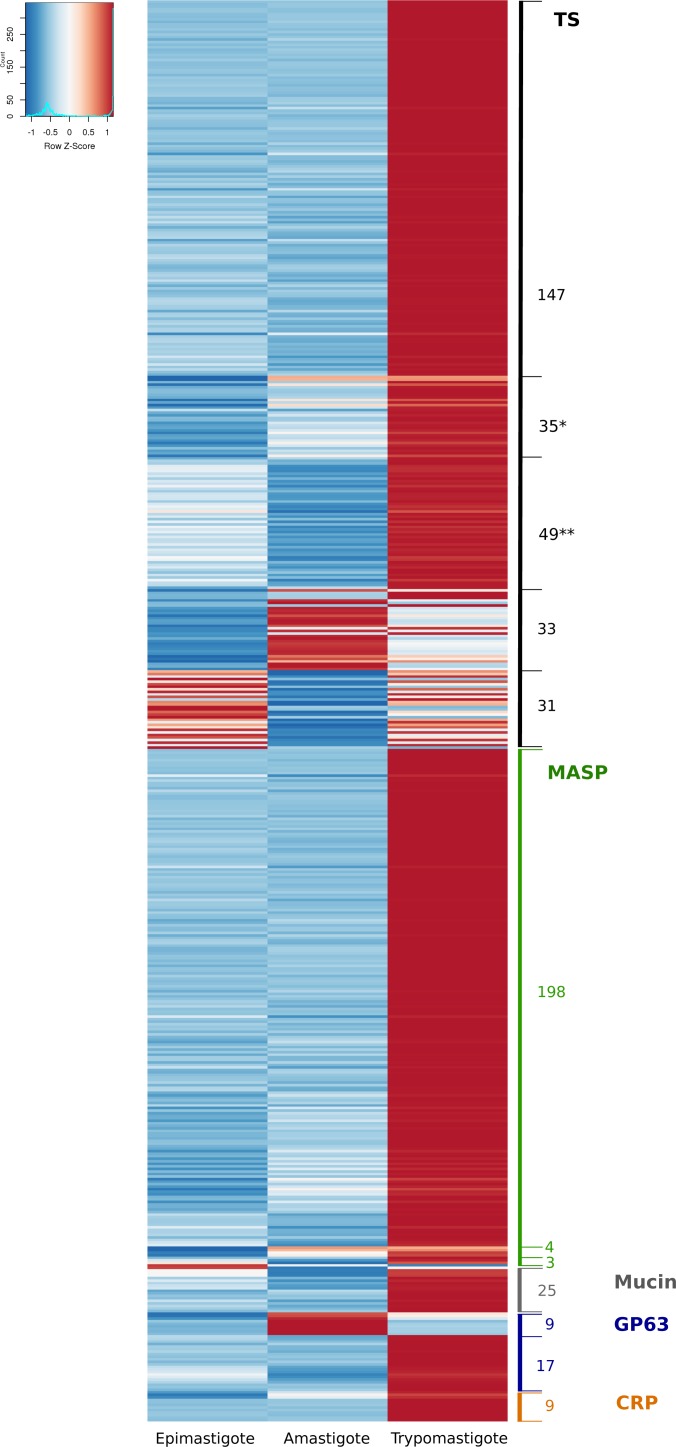
Differential expression of the surface components. Heatmap of glycoprotein genes significantly regulated during the life-cycle of *Trypanosoma cruzi.* The z-score is plotted, red bars represent up-regulation and blue bars represent down-regulation. Numbers correspond to number of genes. (*) remarks TS genes barely expressed in epimastigotes. (**) remarks TS genes barely expressed in amastigotes. A total of 31 and 33 TS genes were up-regulated in epimastigotes and amastigotes, respectively. Of these, 19 and 18 genes were also up-regulated in trypomastigotes. The total number of Trans-sialidases up-regulated in trypomastigotes is 268.

The surface of *T. cruzi* is covered by a dense glycocalix and its composition is characteristic of each differentiation stage, being most of these glycoproteins attached to the plasma membrane by a glycosylphosphatidyl inositol (GPI) anchor (De Lederkremer & Agusti, 2009). Most of the surface proteins belong to multigene families and are involved in the interaction with their hosts (De Pablos & Osuna, 2012). Several studies have been performed in the different protein groups aimed to shed light on their structure, post-translational modifications, their role in the infection and prevalence and the importance as markers or possible drug targets (Acosta-Serrano, Cole & Englund, 2000; Buscaglia, Campo & Frasch, 2006; De Pablos & Osuna, 2012; Freitas et al., 2011; Kawashita et al., 2009). The first *T. cruzi* genome studies have given a more integrative view of the complexity of these expanded families, and have even allowed the identification of a new protein family named Mucin-associated surface proteins (El-Sayed et al., 2005; Franzén et al., 2011; Franzén et al., 2012; Grisard et al., 2014). The main multigene families correspond to trans-sialidases (TS), mucins (MUC), mucin-associated surface proteins (MASP), dispersed gene family-1 (DGF-1) and metalloproteases (GP63). In order to further analyze these families and their expression, we first performed a genomic analysis using public data available in the Tritryp database that includes four *T. cruzi* genomes. Table 3 shows the number of members of these multigene families (including pseudogenes) in different strains analyzed. As it can be observed, the strains exhibit substantial differences in the content of the multigene families. This variation in the membrane composition can constitute a characteristic of the different phylogenetic groups. We further investigated the transcript levels of these gene families during the life cycle of the parasite, and we found that 560 genes encoding surface proteins are differentially expressed along the life cycle, most of them (499) up-regulated in trypomastigotes (Table 3 and Fig. S2). This result is in line with previous reports indicating that most of the members of these families are relevant in the infective stages of the parasite (De Pablos & Osuna, 2012) and also with the enhanced expression of specific enzymes for *O*-glycosylation in this stage (Chiribao et al., 2012). However, a more in-depth analysis of surface multigene families shows that all of them have stage specific genes and some genes are not expressed at all (Fig. 1).

**Table 3:** Membrane components in *T. cruzi* and those differentially expressed in Dm28c.

	TS	MASP	Mucin	DGF-1	GP63	CRP
CL Brener Genome						
Brener	419	345	230	205	93	1
Non-Esmeraldo	579	501	321	186	160	2
Esmeraldo-like	526	531	339	174	172	0
*T. cruzi marinkellei*	841	337	69	709	129	15
Sylvio	1,112	249	76	984	126	4
Dm28c	659	311	116	69	60	13
**DEG**	295 (45%)	205 (66%)	25 (22%)	1 (1%)	26 (43%)	9 (69%)
Up-regulated in trypomastigote	268 (92%)	198 (97%)	25 (100%)	0 (0%)	10 (38%)	9 (100%)

#### Trans-sialidases (TSs)

TSs were described as the largest gene expansion phenomena in the CL Brener strain of *T. cruzi* (El-Sayed et al., 2005), and as we show in Table 3, the analysis of all currently available genomes confirms that it constitutes a general phenomena in *T. cruzi*. An interesting observation from the analysis of TSs expression is that all of them are regulated during the life cycle of the parasite (Fig. 1): 268 genes (80%) are up-regulated in trypomastigotes, but 33 genes are almost exclusively expressed in amastigotes, whereas 31 genes are up-regulated in epimastigotes (Fig. 1). Moreover the figure evidenced two groups of TS genes that are up-regulated in trypomastigotes but clearly appear switched off in epimastigotes (* in Fig. 1), or switched off in amastigotes (** in Fig. 1). It has been well established the relevance of TSs in the infective stages of *T. cruzi*, due to their participation in adhesion and invasion of host cells through different functions. In the first place, *T. cruzi* is unable to synthesize sialic acid, and relies for its acquisition on the surface TSs. TSs are capable of transferring sialic acid residues from host sialoglycoconjugates to parasite mucins (Ferrero-García et al., 1993; Frasch, 2000; Schenkman et al., 1991). Second, these proteins participate in the recognition process through binding to specific receptors (laminin, Trk, among others), and it has been demonstrated that the different variants could explain at least in part the organ tropism of these parasites (Magdesian et al., 2001; Tonelli et al., 2010). Finally, non-infective epimastigotes also express functional trans-sialidases, and, despite their role is unclear, it is accepted that they can participate in insect-parasite interactions and metacyclogenesis (Chaves, Briones & Schenkman, 1993).

The described functions of TSs are clearly related to invasion, escape from parasitophorous vacuole, modulation of immune response and apoptosis and hence the predominance of TSs in trypomastigotes. Nevertheless, some TS members are specifically expressed in amastigotes and epimastigotes. To analyze the characteristics of these groups, we conducted comparisons among the stage-specific up-regulated TSs, namely those exclusively expressed in epimastigotes (eTS), in trypomastigotes (tTS) or in amastigotes (aTS). We compared among these groups of proteins basic features such as predicted GPI anchor addition signal and estimated molecular weight. Our results show that eTS and aTS are smaller than tTS, the mean predicted molecular weight were 27,7 KDa for eTS, 55,3 kDa for aTS and 67,6 kDa for tTS (Fig. S1). Protein characterization showed that eTS are around 60 kDa and tTS molecular weights ranging from 120 to 240 kDa (Briones, Egima & Schenkman, 1995; Schenkman et al., 1991). These differences may be attributed to oligomer formation but also to glycosylation, which are not included in our prediction. In addition, analysis of GPI anchor prediction revealed that none of eTS contain predicted GPI anchor sites (100%), 43% of aTS contain predicted GPI sites whereas 60.6% of tTS are probably GPI anchored. These results support previous reports showing that only 70% of GPI anchored TSs are released after phospholipase C treatment in trypomastigotes (Rosenberg et al., 1991), and also the fact that eTS are not released after addition of phospholipase C (Rosenberg et al., 1991).

Complement regulatory proteins (CRP) constitute a subgroup belonging to the TS superfamily that were analyzed separately for two reasons: first, their relevant role restricting the activation of the complement pathway and the lysis of the parasite (Norris, 1998) (a resistance function that in *Leishmania* is mediated by one group of GP63 proteases (Grandgenett et al., 2000) and second because we found that, like strain Sylvio X10/1, Dm28c has 13 CRP genes whereas in other strains they are single (or few) copy genes (Table 3). Our analysis showed that all CRP genes were significantly over expressed in trypomastigotes, and even more, 70% were almost exclusively expressed in trypomastigotes (Fig. 1 and Table S2). It was previously reported that CRPs are expressed in metacyclic and cell derived trypomastigotes surface, but they are undetectable in epimastigotes and amastigotes (Norris, 1998). Our results show a correlation between protein and mRNA levels for this gene family, and also confirm their specificity for trypomastigotes as expected considering their function. Further studies comparing complement-mediated lysis between other *T. cruzi* strains can give clues about correlation between this group of genes, complement resistance and infectivity.

#### Mucin-associated surface proteins (MASPs)

MASP genes were up-regulated in trypomastigotes (97%) which confirm previous studies obtained using 3′UTR as a probe in Northern blot experiments in the CLBrener strain (Bartholomeu et al., 2009). However, a discrete number of genes were found as differentially up-regulated specifically in amastigotes or epimastigotes (Fig. 1 and Table S2). Being the second largest multigene family in *T. cruzi*, MASPs were described for the first time after sequencing the *T. cruzi* genome (El-Sayed et al., 2005), and their precise function and expression remains unclear Differences in genes up-regulated in specific stages of the parasite deserve further investigation, which can help to unravel the precise function of MASP family components.

#### Mucins (MUCs)

MUCs, also named mucin-like proteins, are the major component of the *T. cruzi* surface. They play different roles according to the stage and environment; in epimastigotes they are smaller, more conserved and participate in the adhesion to the perimicrovillar membrane in the insect intestine. They also confer protection against proteolysis (Buscaglia, Campo & Frasch, 2006). Mucins expressed in trypomastigotes have high glycan content and diversity, higher molecular weights, and their roles are related to attachment and penetration as well as immune evasion (Buscaglia, Campo & Frasch, 2006). Although our analysis reveals that only 25 differentially expressed mucin genes are up-regulated in trypomastigotes in comparison with both amastigotes and epimastigotes (fold change greater than two and FDR < 0.05, Fig. 1), the rest of the them, are expressed at higher levels in trypomastigotes: all TcMUCI (19 genes) and 52 out of 72 TcMUCII (Table S9). On the one hand, the fact that most of the TcMUCII are highly expressed in trypomastigotes is in agreement with previous experiments showing that this subgroup is the preferred mucin at this stage (Buscaglia, Campo & Frasch, 2006). On the other hand, the highest expressed mucins in amastigotes do not belong to TcMUCI group but TcMUCII, in disagreement with previous immunofluorescence experiments showing TcMUCI as the predominant group at this stage (Buscaglia, Campo & Frasch, 2006). Two facts are worth stressing: first, only 19 genes have been identified in Dm28c so far (a reduced number in comparison with other available genomes), so we cannot rule out the possibility of not being appreciating the full picture. Second, all TcMUCI genes have a moderate expression, lower in average than that of TcMUCII for all stages. Finally, there is also a group of 23 genes that drew our attention because they present very low or almost no expression in all three stages. The reason why these genes are turned off in the three stages analyzed escapes our knowledge. Additional studies on the expression of these and other membrane proteins are essential to shed light on these topics.

#### GP63

GP63 proteins are surface GPI anchored metalloproteases present in *Leishmania spp.*, African trypanosomes and *T. cruzi* (Cuevas, Cazzulo & Sánchez, 2003; LaCount et al., 2003). In *Leishmania* species GP63 proteins and their coding genes have been extensively studied (evolution, organization of multigene family and its role in invasion,) (Yao, Donelson & Wilson, 2003). However in *T. cruzi* little is known about these genes. As it is clear from Table 3, most available genomes of *T. cruzi* contain about 170 genes per haploid genome, but in Dm28c there are only 60 annotated GP63 genes. The expression analysis of these genes allowed us to divide them into two groups: those that were found to be barely expressed in all stages of the parasite (50%), and a second group of genes that are differentially expressed (Fig. 1 and Fig. S3). This latter, in turn, can be divided into two sub-groups: a first one significantly up-regulated in trypomastigotes, and a second subgroup up-regulated in amastigotes (containing 17 and 9 genes respectively). This suggests a fine regulation of the steady state levels of their mRNAs. Grandgenett and collaborators have suggested dividing the family in two categories according their expression in different stages and the length of their 3′UTRs (Grandgenett et al., 2000). Subsequently, Cuevas and co-workers defined again two subgroups: Tcgp63-I that is widely expressed and Tcgp63-II that is scarcely detected in Northern blot analyses (Cuevas, Cazzulo & Sánchez, 2003). Our results, which are in line with these previous works, give now a more complete view of the expression pattern of all the GP63 family, indicating a relevant role in the mammal stages of the parasite.

**Figure 2:**
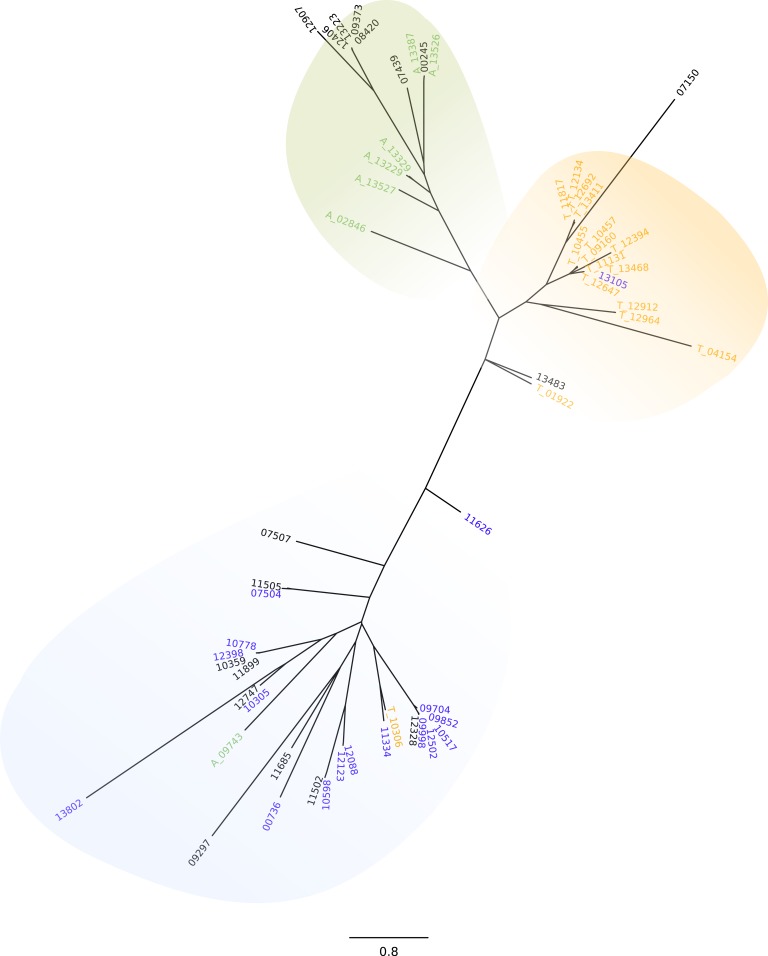
GP63 3′UTR phylogeny. Neighbor-joining tree of the 3′UTR of GP63 genes; numbers correspond to gene ID in Dm28c annotation. Differentially up-regulated in A (green), differentially up-regulated in T (orange), very low or null levels of expression (blue).

Due to the probable relevance of UTR regions in stage specific expression regulation (Grandgenett et al., 2000), and taking into account the differences in sequence and length of the ’UTR, we further investigated the GP63 genes and their 3′UTRs. We first conducted a phylogenetic analysis of the amino acid sequences. By doing this we could observe that all GP63 genes that are DEGs in amastigotes clustered together and the same was true for GP63 genes that were up-regulated in trypomastigotes with the exception of 3 sequences (Fig. S3). More interesting though, are the results from the analysis of the 3′UTR, which show three groups of sequences clearly differentiated: those that belong to isoforms highly expressed in amastigotes, those associated to genes highly expressed in trypomastigotes and those associated to the group of genes with almost no expression in any stage of life cycle (Fig. 2). The fact that each main group of 3′UTR is associated to a specific stage of the life cycle is a strong indication of the relevance of 3′UTR in post transcriptional regulation. Whether this is due to the presence of sequences or motives that either stabilize or degrade GP63 mRNA differentially during life cycle is uncertain. Further work will be necessary to identify mRNA-conserved motives in these genes and RNA binding proteins or small RNAs responsible for this tight regulation.

#### Dispersed gene family 1

Located in sub-telomeric regions, this group of proteins is greatly expanded in CLBrener strain where they are divided in at least 3 groups (Kawashita et al., 2009). However, the first aspect that attracted our attention of this family is its reduction in the Dm28c strain, which contains only 69 annotated gene copies (Table 3). Taking into consideration that several genes appear to be incomplete (since they exhibit a reduction in length to less than 3,000 bp, when the estimated size is around 10 kb) the low number of DGF-1 genes in Dm28c most likely is the consequence of inaccurate genome assembly. Previous studies detected DGF-1 gene expression in different stages (Kawashita et al., 2009). Moreover, proteomic analyses find DGF-1 proteins in the parasite surface (Atwood et al., 2006). Our results do not show differential expression of these genes among the different three stages analyzed, but it should be pointed out that there is a group of DGF-1 genes that are almost not expressed. Nevertheless these results deserve further investigation because the incomplete annotation of this group could lead to erroneous conclusions.

The results obtained in this study, concerning the stage specific membrane composition variation and the surface remodeling during stage transitions, are depicted in Fig. S2. This figure summarizes the expression levels of each gene belonging to surface multigene families in the different stages and highlights the stage-specific genes (Fig. S2A); The general picture of expression of these families (i.e., total read counts of each family in the three stages) is also represented (Fig. S2B).

**Figure 3:**
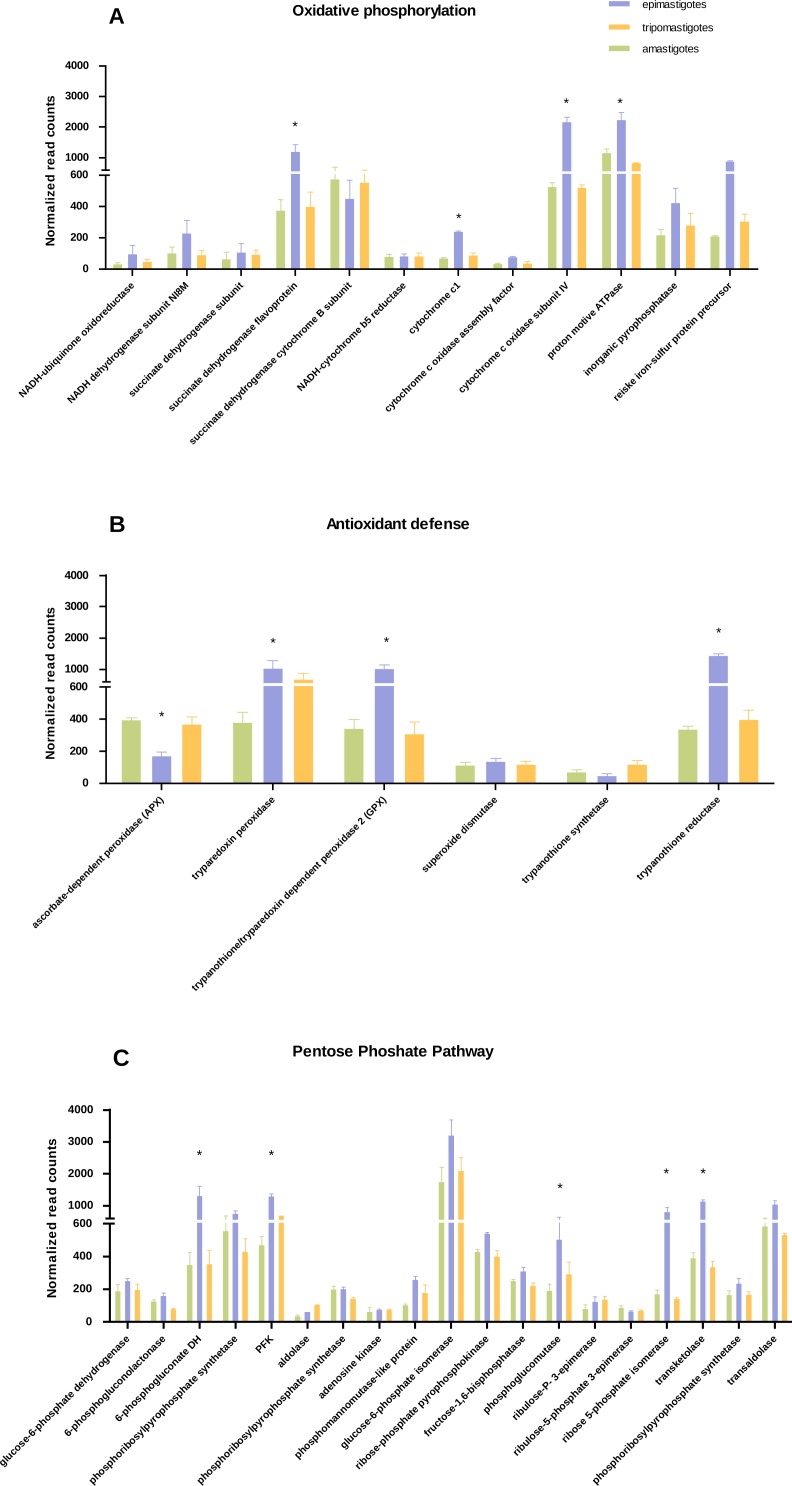
Differential expression in metabolic pathways. Normalized read counts for each stage is shown for (A) Oxidative phosphorylation; (B) Antioxidant defense; and (C) Pentose phosphate pathway. Different cycle stages are represented: amastigotes (green), epimastigotes (blue) and trypomastigotes (orange). (*) Denotes differentially expressed genes.

### Metabolic switch through *T. cruzi* life cycle

#### Oxidative metabolism

We have found that genes belonging to Krebs cycle, respiratory chain and oxidative phosphorylation, were significantly up-regulated in epimastigotes, suggesting an enhanced respiratory activity at this stage (Fig. 3A and Table S3). However expression of genes related to respiration was detected in all stages. Analysis of respiratory chain genes showed very low expression of complex I associated components (NADH dehydrogenase subunit, NADH-ubiquinone oxidoreductase) in all samples (Table S3). This result supports the notion that complex I is not very active in *T. cruzi* as was suggested previously (Carranza et al., 2009). On the other hand, the relevance of succinate-dependent respiration was evidenced here by the up-regulation of NADH dependent fumarate reductase in epimastigotes, which generates succinate as the main source of electrons in the respiratory chain (Denicola-Seoane et al., 1992). The increase in oxidative metabolism in epimastigotes is in concordance with its high anabolic profile, which suggests that in this stage synthesis of macromolecules and particularly of steroids is favored.

Regarding the increase in respiration related genes, we wondered if the expression of antioxidant enzymes and NADPH production coding genes were also up-regulated, as a strategy to avoid oxidative damage. Analysis of antioxidant genes also showed a general up-regulation of many genes that participate in antioxidant defenses in epimastigotes (trypanothione reductase, GPX, and tryparedoxin peroxidase) that could play a role against reactive oxygen species produced by high respiratory chain activity (Fig. 3B and Table S4). In contrast, ascorbate dependent peroxidase (APX) is up-regulated in mammalian stages (trypomastigotes and amastigotes). APX uses ascorbate as electron donor (Logan et al., 2007), and it has been proposed that in both stages *T. cruzi* is able to synthesize this vitamin (Logan et al., 2007). In this context, the down-regulation of APX in epimastigotes might reflect the inefficiency in ascorbate synthesis and/or reduction in this stage. The reduced levels of APX mRNA genes is also in line with the empirical observation that ascorbic acid content in epimastigotes is between 1,6 and 3,6 times lower than trypomastigotes (Clark, Albrecht & Arevalo, 1994).

Concerning the expression of antioxidant genes, pentose phosphate pathway genes were also up-regulated in epimastigotes (Fig. 3C and Table S6), suggesting not only a greater production of NADPH, which acts as an electron donor in detoxifiying reactions, but also a production of ribose phosphate for nucleotide synthesis.

Another remarkable point is that enzymes for fermentation (acetaldehyde dehydrogenase and alcohol dehydrogenase) were highly up-regulated in epimastigotes. These results show that during this stage of life cycle the parasites can be adapted to different metabolic conditions, particularly to different oxygen conditions for ATP production.

#### Lipid metabolism

**Figure 4:**
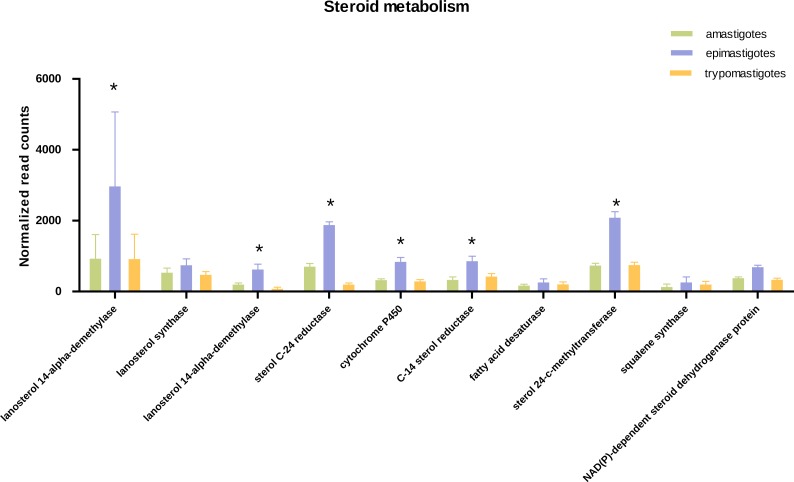
Expression of steroid biosynthesis related genes. Total normalized count of reads is shown for steroid metabolism genes for the three stages: amastigotes (green), epimastigotes (blue) and trypomastigotes (orange). (*) Denotes differentially expressed genes.

Analysis of genes related to lipid metabolism in *T. cruzi* main stages showed significant differences. Both epimastigotes and amastigotes (compared to non-dividing trypomastigotes) up-regulate key genes involved in phospholipid and sterol biosynthesis (Fig. 4 and Table S5). Comparison of genes related to lipid synthesis in amastigotes and epimastigotes revealed that the former not only have higher mRNA levels of genes involved in phospholipid synthesis (phosphatidic acid phosphatase, choline ethanolamine kinase) but also of desaturases which allow the generation of polyunsaturated fatty acids that maintain membrane fluidity under variable environment (Table S5).

Concerning sterol biosynthesis pathways it was demonstrated that in amastigotes it is similar to epimastigotes but simpler, being cholesterol (probably derived from the host) up to 80% in weight of total sterols (Liendo et al., 1999). Analysis of sterol synthesis genes showed an up-regulation in most genes of this pathway in epimastigotes (Fig. 4). This may constitute an advantage due to the variable temperature in the insect host and the subsequent requirement to regulate membrane fluidity. In this sense, it was shown that epimastigotes have the ability to rapidly adapt and remodel their lipid content in response to temperature changes (Florin-Christensen et al., 1997).

We have also analyzed the expression of genes related to catabolism of fatty acids and found that all stages have similar levels of mRNAs encoding beta oxidation enzymes, although some genes relevant for activation and transport of fatty acids exhibited increased mRNA levels in amastigotes and epimastigotes (fatty acyl CoA synthase, carnitine *O*- acyl transferase). Down-regulation of fatty acid oxidation related genes in trypomastigotes was also observed by Li and collaborators (2016) during the transition from trypomastigotes to intracellular amastigotes. Atwood and co-workers (Atwood et al., 2006) suggested a shift from carbohydrate to fatty acid catabolism in the transition of trypomastigotes to amastigotes, on the basis of proteomic data that showed the presence of *β* oxidation enzymes as well as Krebs cycle intermediates. In contrast, metabolic studies confirm that amastigotes can use glucose as a carbon source generating acetate, glycerol and pyruvate (Sanchez-Moreno et al., 1995). It is important to note that metabolic, transcriptomic and proteomic studies had been carried out using *in vitro* approaches, sometimes differing from real physiological and environmental conditions. Our data shows that epimastigotes, amastigotes and trypomastigotes express high levels of *β* oxidation related genes, but some of them, like acyl CoA dehydrogenase and enoil CoA isomerase are up-regulated in epimastigotes (Table S5). Comparison of amastigotes and trypomastigotes revealed that fatty acid oxidation genes (ketoacyl-CoA thiolase, enoyl-CoA hydrtase) were overexpressed in the former, as in amastigotes of the Y strain (Li et al., 2016), suggesting that it constitutes a general feature of amastigotes, independently of the lineage.

#### Glucose catabolism

**Figure 5:**
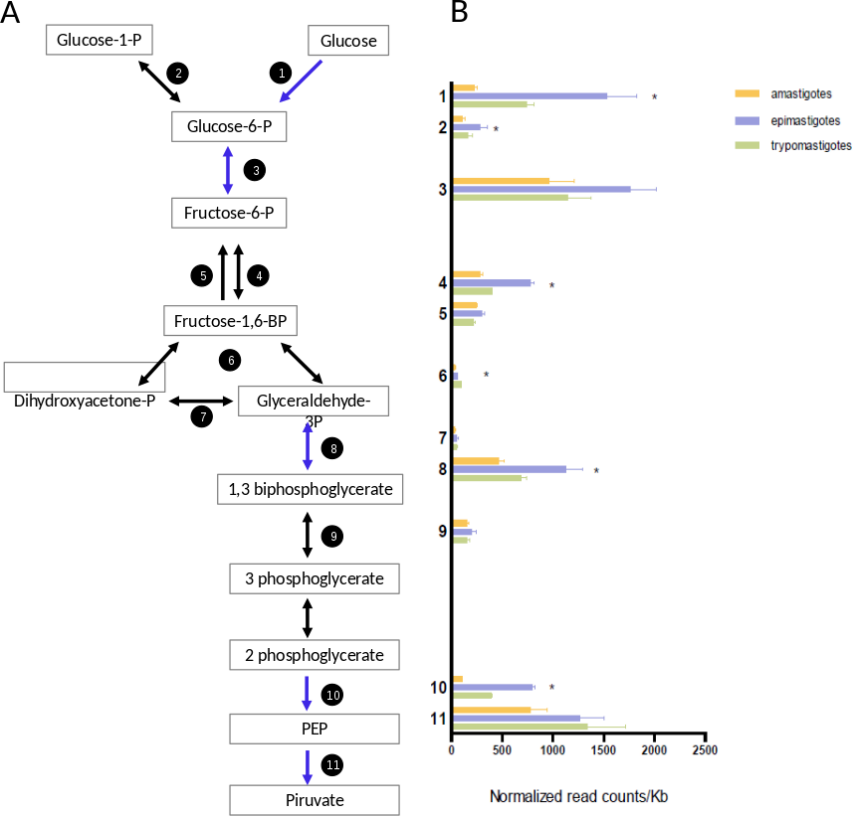
Glucose metabolism. (A) Schematic diagram of glucose catabolism. Each reaction is assigned with a number (1: hexokinase, 2: phosphoglucomutase, 3: glucose-6-phosphate isomerase, 4: phosphofructokinase, 5: fructose-1,6-biphosphatase, 6: aldolase, 7: triosephosphate isomerase, 8: glyceraldehyde 3-phosphate dehydrogenase, 9: phosphoglycerate kinase, 10: enolase and 10: pyruvate kinase 2). (B) Expression of glucose metabolism genes of each reaction is shown as normalized count per gene size in kilobases. The three cycle stages are represented: amastigotes (green), epimastigotes (blue) and trypomastigotes (orange). (*) Denotes differentially expressed genes.

Trypomastigotes are present in the blood of their mammalian host, where glucose is abundant; amastigotes reside in the cytoplasm of mammalian cells where free glucose is scarce; and epimastigotes live in the digestive tract of the insect (an amino acid rich medium), which has sources of free glucose during or immediately after bloodmeals. The transcriptomic profiling of genes encoding glycolytic enzymes showed that all of them are expressed, but epimastigotes present higher mRNA levels of hexokinase, phosphofructokinase, glyceraldheyde-3-phosphate dehydrogenase and enolase than trypomastigotes and amastigotes (Fig. 5 and Table S6). Comparison of normalized mRNA levels (ncounts/Kb) showed significant differences between the genes in the same pathway (Fig. 5). An overview of Fig. 5 shows that the most highly expressed glycolytic genes are in the extremes of the graphics, that is, in the initial and final steps of glycolysis. These are the most relevant enzymes since they catalyze either the points of regulation of the pathway and/or reactions related to ATP production. It is well known that intermediate reactions depend on the availability of substrates, and they do not need to have high levels of expression, this is the case of genes 4, 6, 7 and 9 (Fig. 5). Glyceraldehyde-3-phosphate deydrogenase (GAPDH) constitute an exception since their level of transcription is higher. However, it should be noted that this gene encodes cytosolic, instead of glycosomal enzyme and probably high concentrations are required due to the lack of compartmentalization. On the other hand, the GAPDH reaction is responsible for the first “high energy” intermediate formation and therefore it is a hub for ensuring metabolic flux of the pathway. In summary, although epimastigotes present higher level of some key glycolytic genes, all the stages are prepared for glucose degradation. Additionally, we cannot discard that these differences in mRNA levels could also be a strategy for glycolysis regulation under different stimuli like hypoxia or glucose availability. This kind of regulation has been observed in yeast (Daran-Lapujade et al., 2007), were post-transcriptional regulation plays major roles in modulating metabolism.

A particular highlight of our results is that amastigotes present a drastic reduction of hexokinase (HK) mRNA levels (almost seven and three times lower than epimastigotes and trypomastigotes respectively, see Fig. 5). It has been well established that amastigotes use mainly amino acids as primary source of energy (Silber et al., 2005), and the drastic down-regulation of HK expression could imply a reduction of glycolysis. In fact, glucose transporters are not expressed in this stage (Silber et al., 2009) in agreement with the low intracellular glucose concentration of around 20 µM (Malliopoulou et al., 2006). However, the rest of the glycolytic enzymes do not decrease their expression, suggesting that the pathway could be active in the presence of hexose phosphates, but specific transporters have not been described in *T. cruzi*. Therefore, the HK decrease can reflects a switch to gluconeogenesis at this stage. It should be noted that, as mentioned above, amastigotes express high levels of genes related to pentose pathway, including those coding for the non-oxidative phase of the pathway (Fig. 3C). These enzymes are responsible for the interconversion of monosaccharide-phosphates from 3 to 7 C, then generating hexose phosphates as substrates for glycolysis.

Finally we have found that genes encoding enzymes necessary for fermentation (acetaldehyde dehydrogenase and alcohol dehydrogenase) were highly up-regulated in epimastigotes. These results show that they are adapted to different metabolic conditions, as well as to different oxygen conditions for ATP production. It is known that trypanosomatids produce and excrete reduced fuels, not only in anaerobiosis, but also in the presence of oxygen (Cazzulo, 1992). Interestingly, Sanchez-Moreno and coworkers have showed that epimastigotes but not amastigotes, release ethanol to the media using glucose as a carbon source (Sanchez-Moreno et al., 1995), which is in line with our findings.

#### Protein and amino acid metabolism

Protein synthesis and degradation plays important roles in parasites, which suffer morphological changes and nutritional stresses through their life cycle. Synthesis of new specialized proteins and glycoproteins is necessary for adaptation and survival in each stage. Furthermore, proteins and amino acids can be used as major carbon sources for ATP production depending on the stage and/or the environment (Cazzulo, 1984).

In *T. cruzi*, amino acid catabolism is very relevant in proliferative stages, amastigotes use amino acids for energy production and epimastigotes also use amino acids when glucose is not available (Bringaud, Riviere & Coustou, 2006). In addition, some amino acids like arginine and proline play additional roles like energy store and differentiation respectively (Silber et al., 2005). Besides, proline has an outstanding role promoting the differentiation of intracellular forms, from epimastigotes-like to trypomastigotes (Tonelli et al., 2004) as well as in metacyclogenesis (Homsy, Granger & Krassner, 1989).

**Figure 6:**
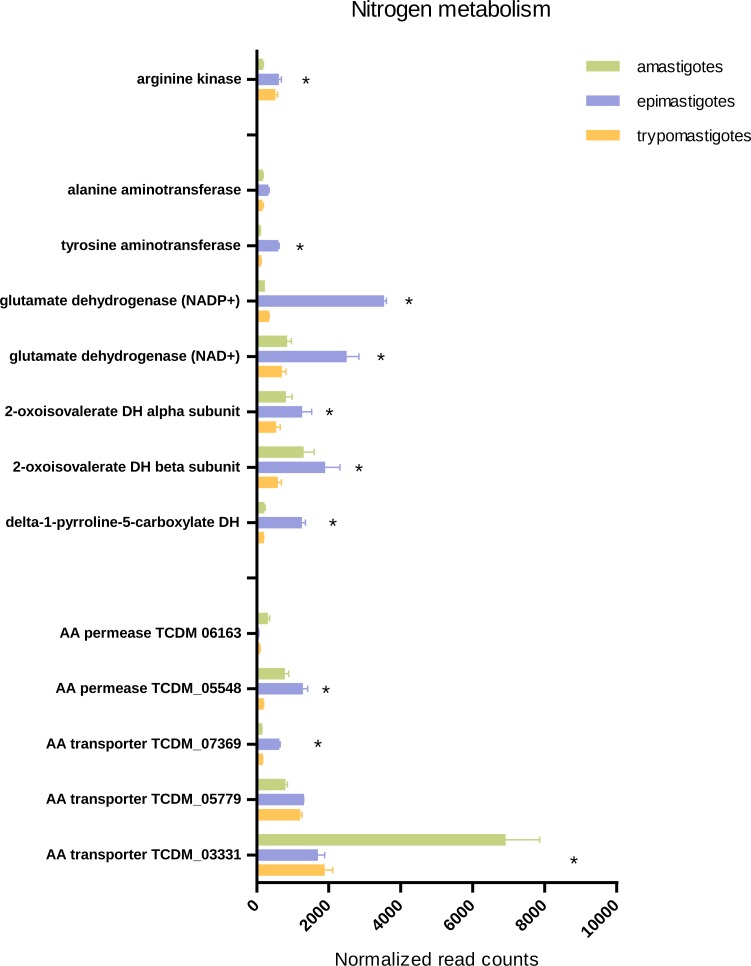
Expression of genes related to amino acid transport and metabolism. Total normalized Read counts of some genes coding amino acids transporters and nitrogen and amino acid related metabolism. The three cycle stages are represented: amastigotes (green), epimastigotes (blue) and trypomastigotes (orange). (*) Differentially expressed.

Our results emphasize the significance of amino acids in *T. cruzi* biology and metabolism as indicated by the high expression of several amino acid permeases and transporters in epimastigotes, trypomastigotes and amastigotes. Our transcriptomic analysis revealed that each stage presents specific highly expressed amino acid transporters, even trypomastigotes. Nevertheless most transporters were up-regulated in amastigotes and epimastigotes, suggesting that amino acid metabolism is more relevant in proliferative stages than in trypomastigotes (Fig. 6 and Table S7).

It has been demonstrated that epimastigotes can metabolize asparagine, aspartate, glutamine, glutamate and branched amino acids like valine, leucine and proline, and their oxidation converges in aspartate and glutamate. Glutamate can participate as substrate of transaminases or deaminases and enter the Krebs cycle (Silber et al., 2005). To check these aspects we looked for genes involved in valine, leucine and isoleucine degradation and their expression through the life cycle. Our data agree with the idea that catabolism of these amino acids is increased in epimastigotes since most genes of this pathway were up-regulated in this stage. It should be pointed out that one of the most important enzymes involved in branched amino acid degradation, the oxoisovalerate dehydrogenase complex, is also up-regulated in amastigotes (Fig. 6, Table S7).

The amino group of glutamate can be transferred to pyruvate by transaminases (alanine aminotransferase or tyrosine aminotransferase) or alternatively transferred to water by glutamate dehydrogenases, releasing NH_3_. In this context, we looked for glutamate dehydrogenases coding genes (NADP+ and NAD+ dependent) and tyrosine and alanine aminotransferases, and it was found that both glutamate dehydrogenases were up-regulated in epimastigotes; in particular, NADP+ dependent glutamate dehydrogenase mRNA levels increased 10 fold in this stage (Fig. 6, Table S7). All these results strongly support the idea that amino acid and nitrogen metabolism is enhanced in epimastigotes due to the scarcity of carbohydrates and the abundance of proline in the terminal portion of the digestive tube of the triatomine (Manchola et al., 2016). In this sense, a general down-regulation of processes related to amino acid metabolism and transport was observed in trypomastigotes, supporting the idea that amino acids are not the preferred fuel when they parasite the mammal host (Bringaud, Riviere & Coustou, 2006).

As mentioned above, arginine participates in energy storage through a reaction catalyzed by arginine kinase. This reaction generates phosphoarginine, which serves as an ATP, and phosphate reservoir and supports burst of cellular activity during the life cycle (Alonso et al., 2001). Our results show that this gene is significantly up-regulated in epimastigotes and trypomastigotes; and this might reflect the fact that amastigotes have a constant supply of glucose and amino acids and therefore do not need such energy storage. Another explanation is that arginine kinase activity was not directly correlated with mRNA levels, a result already shown for epimastigotes (Alonso et al., 2001).

Finally, expression analysis of genes related to proteasomal degradation during the cycle showed that 24 genes coding proteasome subunits are highly expressed during the cycle (Table S7), this is not surprising since parasites undergo radical morphological changes which are carefully controlled by proteasome mediated proteolysis (Munoz et al., 2015).

### Annotation of new genes

During the analysis we found that some genes were not annotated in the Dm28C strain. Additionally the visual inspection of mapped reads evidences transcriptional activity in regions that were annotated as intergenic and also encompass relatively long (>300 nt) open reading frames (ORFs). These three facts led us to look for possible non annotated genes. For this purpose we assembled the transcriptome including all reads from the different libraries. From this assembly we identified 9521 novel transcripts (not coincident with the known annotation) containing 1400 ORFs regions with a minimum length of 300 bp. These ORFs were subsequently validated by HMM and Blast searches against public databases giving a total of 858 new possible coding sequences. These predictions correspond to genes or gene segments that were not annotated in the Dm28c strain. To identify ORFs with higher chances of corresponding to complete CDSs (within this population of non annotated genes) we decided to use a more stringent criterion in this annotation step and kept only those ORFs that align with an annotated protein entry with the following requirements: minimum alignment identity: 60%, minimum query alignment length 60% and, minimum subject alignment length 60%. With this procedure, we identified 247 putative proteins that were not previously annotated in Dm28c. They mostly correspond to hypothetical proteins, but also we identified several surface components (TS, mucins, MASP, etc.), retrotransposon hot spot proteins, among other genes (see Table S8). In particular we found the tryparedoxin 1 (TXN I) gene almost identical (99.3% identity) to the *T. cruzi* tryparedoxin CAC85916.1. Overall, these results illustrate the importance of continuing to progress in the annotation process combining different sources of data and manual curation (Table S8).

The expression analyses presented here were further compared to specific protein expression profiles (Fig. S4). These groups of genes are: (i) flagellum associated genes down-regulated in amastigotes which present a small non-emergent flagellum; (ii) genes related to conversion of histidine to glutamate up-regulated in epimastigotes allowing to this stage to take advantage of the abundance of histidine in the gut of its insect vector (Parodi-Talice et al., 2004); (iii) mucins up-regulated in trypomastigotes, being TcMUC II predominant (70%) over TcMUC I (Fig. S4) ( Buscaglia, Campo & Frasch, 2006); In all the above examples we found a correlation between mRNA expression and protein levels, indicating that regulation of gene expression in trypanosomes is multifactorial, and both translation (Smircich et al., 2015) and transcription profiles this work and (Li et al., 2016) are relevant for specific biological functions.

## Conclusions

In this work we conducted a RNA-seq analysis in *Trypanosoma cruzi*, a species of great medical importance since it is the causative agent of Chagas disease. We have sequenced RNA populations from the three stages of the life cycle of the parasite using Illumina technology. This technology in combination with computational tools was used to perform a comparative analysis of gene expression along the life cycle of *T*. *cruzi*.

**Figure 7:**
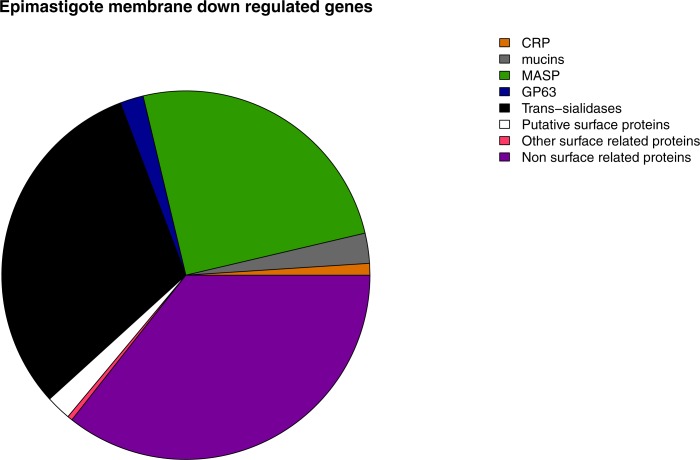
Membrane down-regulated genes in Epimastigotes. Pie chart representation of the percentage of down-regulated genes in epimastigote of each membrane protein category analyzed.

A correlation between patterns of gene expression and previously described metabolic features of each stage was found. Metabolic pathway analysis of highly expressed genes in epimastigotes revealed that they are related to ATP production pathways such as Krebs cycle, pyruvate metabolism, respiratory chain, oxidative phosphorylation and nitrogen metabolism. Biosynthetic pathways related genes are also up-regulated at this stage, being the most important steroid biosynthesis. Gene ontology analysis confirms the pathway enrichment analysis, since the biological processes related to ATP biosynthesis such as carbohydrate and amino acid metabolic catabolism are the most important in epimastigotes, whereas most of the surface genes are down-regulated at this stage (Fig. 7).

**Figure 8:**
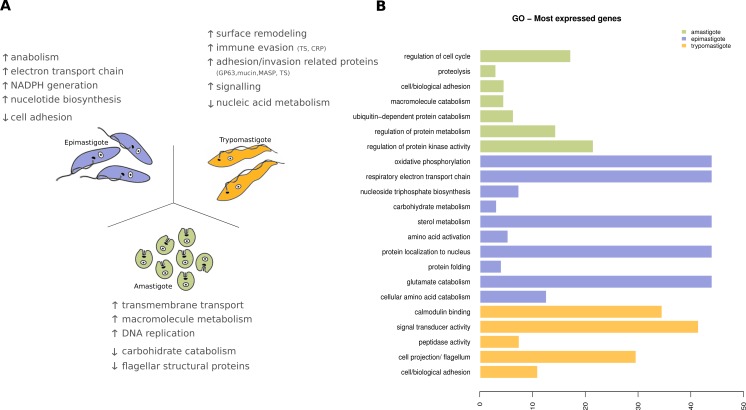
Expression levels overview of Trypanosoma cruzi. (A) Diagram of the *Trypanosoma cruzi* stages and the major findings of transcriptoma analysis. (B) Gene ontology (GO) enrichment analysis, showing GO terms exhibiting statistical significant differences (Fisher Exact Test, filtering *p*-values for multiple testing using False Discovery Rate) for the most expressed genes specific to amastigote (green), epimastigote (blue) and trypomastigote (orange).

In amastigotes (intracellular replicative forms) the highest expressed genes are related to regulation of cell cycle, protein and amino acid catabolic processes, adhesion and signaling. Cell adhesion includes the expression of many surface proteins involved in invasion like stage specific trans-sialidases, GP63, and MASPs. The high expression level of transporters, particularly amino acid transporters, shows that the parasite uses host cytoplasmic metabolites at this stage (Fig. 8). Some of these characteristics were also recently reported for amastigotes in the Y strain (Li et al., 2016) suggesting that the metabolic hallmarks of this stage are independent of the *T. cruzi* lineage considered.

Trypomastigotes (non-replicative and infective form) exhibit a predominance of surface protein genes, those encoding trans-sialidases, MASPs, GP63, mucins and complement regulatory proteins represent more than 50% of the transcripts. Enrichment analysis (GO Biological processes) of these genes revealed that cell adhesion, microtubule-based flagellum, peptidase, signal transducer activity and calmodulin binding are the most relevant ones, in agreement with the specialization of trypomastigotes in movement, adhesion, invasion and signaling (Fig. 8).

A total of 1400 ORF regions with a minimum length of 300 pb were identified, that eventually correspond to 858 new coding sequences. By using stringent matching conditions, 247 proteins were identified as non-annotated in the Dm28c genome.

In summary, transcriptome profiling of the three main developmental stages of *Trypanosoma cruzi* has shown which genes and processes are related to each stage, and allows to conclude that surface remodeling and metabolic switches are at the basis of differentiation.

##  Supplemental Information

10.7717/peerj.3017/supp-1 Table S1 Highly expressed genes and GO analyses Comparison of 500 highest expressed genes among stages and Gene Ontology analysis.

10.7717/peerj.3017/supp-2 Table S2 Differentially expressed genes Result of differentially expression analysis. Gene ontology of stage specific DEGs.

10.7717/peerj.3017/supp-3 Table S3 Oxidative metabolism Normalized read counts of Krebs cycle and respiratory chain genes.

10.7717/peerj.3017/supp-4 Table S4 Antioxidant genes Normalized read counts of antioxidant genes.

10.7717/peerj.3017/supp-5 Table S5 Lipid metabolism Normalized read counts of genes related to different lipid related metabolic processes: fatty acid, steroid and phospholipid.

10.7717/peerj.3017/supp-6 Table S6 Carbohydrate metabolism Normalized read counts of glycolytic and pentose phosphate pathway genes.

10.7717/peerj.3017/supp-7 Table S7 Protein metabolism Normalized read counts of genes coding general protein metabolic pathways including protein degradation, protein synthesis, amino acid transport and metabolism.

10.7717/peerj.3017/supp-8 Table S8 New genes prediction Characterization of putative new transcripts predicted for Dm28c. Statistical description of the validation by HMM and Blast searches.

10.7717/peerj.3017/supp-9 Table S9 Raw and normalized counts Raw transcript levels from ERANGE. Normalized counts were obtained from DESeq2.

10.7717/peerj.3017/supp-10 Figure S1 Stage specific TS examination Representation of predicted molecular weights of trans-sialidases specific from amastigotes (aTS, green), epimastigotes (eTS, blue) and trypomastigotes (tTS, orange). *T* test was performed, ** represents *p*-value <0.01.

10.7717/peerj.3017/supp-11 Figure S2 Surface gene composition in different *T. cruzi* stages (A) Expression of each gene in normalized read counts is shown for the different group of membrane component. At the *X* axis each gene is plotted at the same order, for the three stages. (B) Sum of total normalized read counts per gene Kb of each family group in the three stages. Different cycle stages are represented: amastigote (green), epimastigote (blue) and trypomastigote (orange).

10.7717/peerj.3017/supp-12 Figure S3 GP63 philogeny Neighbor-joining tree of the protein sequences of GP63 genes; numbers correspond to gene ID. Differentially up-regulated in A (green), differentially up-regulated in T (orange).

10.7717/peerj.3017/supp-13 Figure S4 Gene expression patterns Comparison to known protein expression profiles. (A) Flagellum associated genes down-regulated in amastigotes. (B) Genes related to conversion of histidine to glutamate up-regulated in epimastigotes. (C) Mucins up regulated in trypomastigotes.

## Additional Information and Declarations

### DNA Deposition

The following information was supplied regarding the deposition of DNA sequences: Raw sequences were deposited at the NCBI database under SRA accession number SRP072022.

### Data Availability

The following information was supplied regarding data availability: The raw data included in the manuscript are the count of mapped reads in each transcript. The raw counts and normalized counts of transcripts are presented in Table S9.
