# Supplementary figures and images for: Transcriptomic analysis reveals metabolic switches and surface remodeling as key processes for stage transition in Trypanosoma cruzi
Source: PeerJ. 2017 Mar 8;5:e3017. doi: 10.7717/peerj.3017 (PMC5345387; doi:10.7717/peerj.3017)

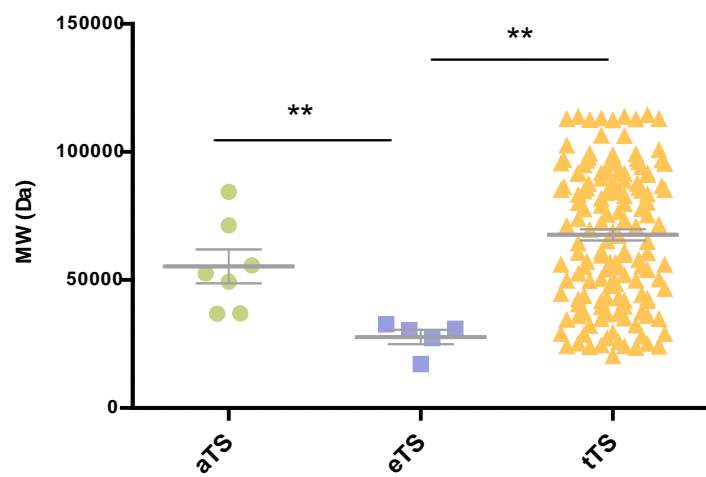

Supplement: Figure S1 — Representation of predicted molecular weights of trans-sialidases specific from amastigotes (aTS, green), epimastigotes (eTS, blue) and trypomastigotes (tTS, orange). T test was performed, ** represents p-value <0.01. [file peerj-05-3017-s010.pdf]

A

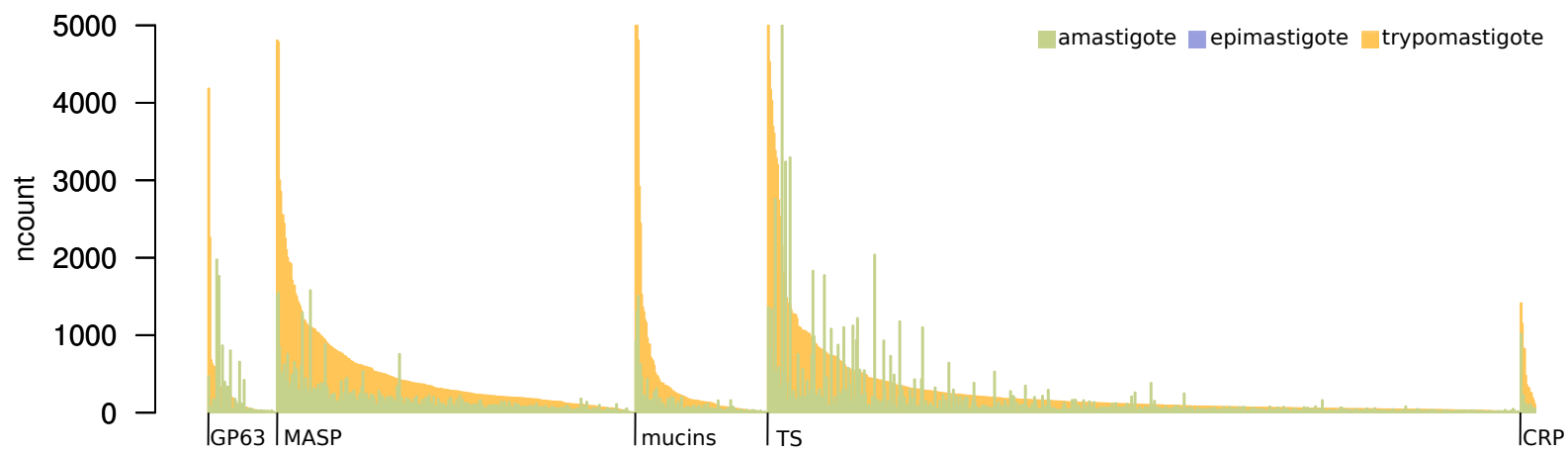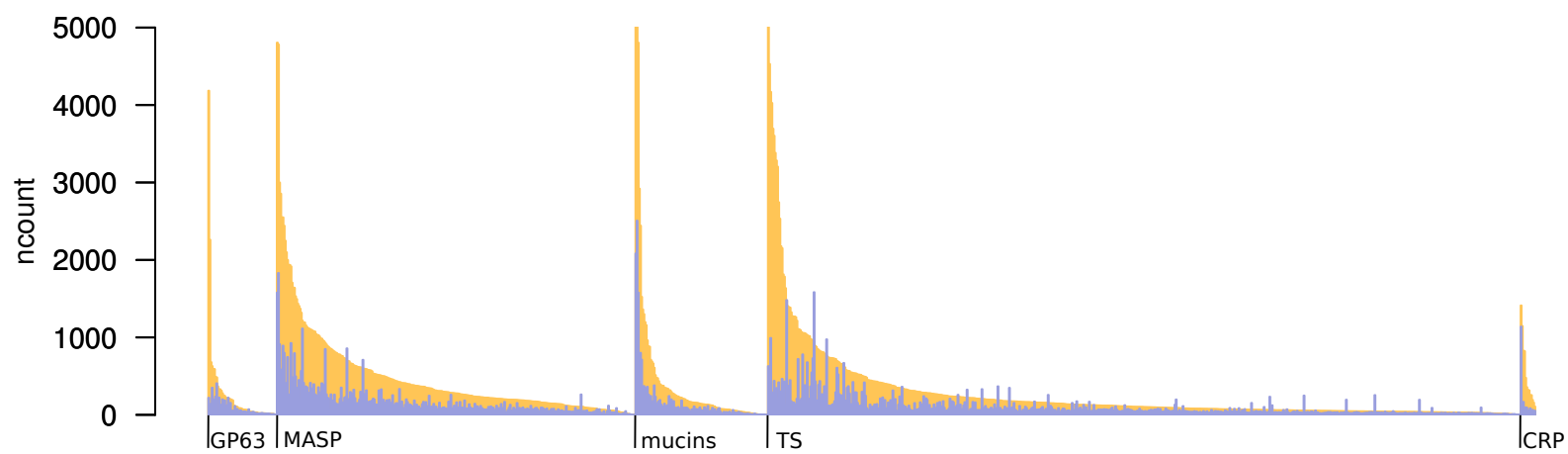

B

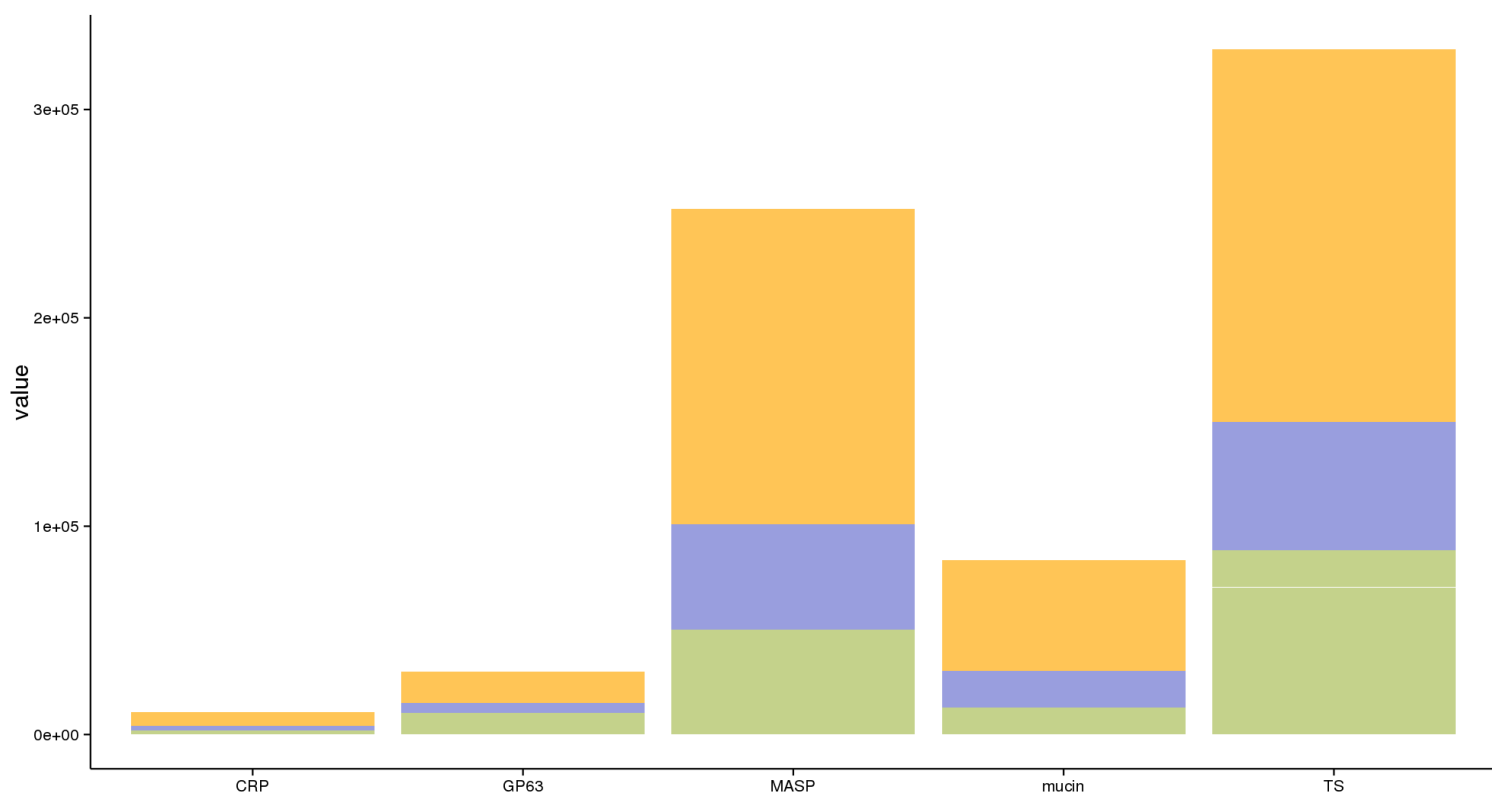

Supplement: Figure S2 — (A) Expression of each gene in normalized read counts is shown for the different group of membrane component. At the X axis each gene is plotted at the same order, for the three stages. (B) Sum of total normalized read counts per gene Kb of each family group in the three stages. Different cycle stages are represented: amastigote (green), epimastigote (blue) and trypomastigote (orange). [file peerj-05-3017-s011.pdf]

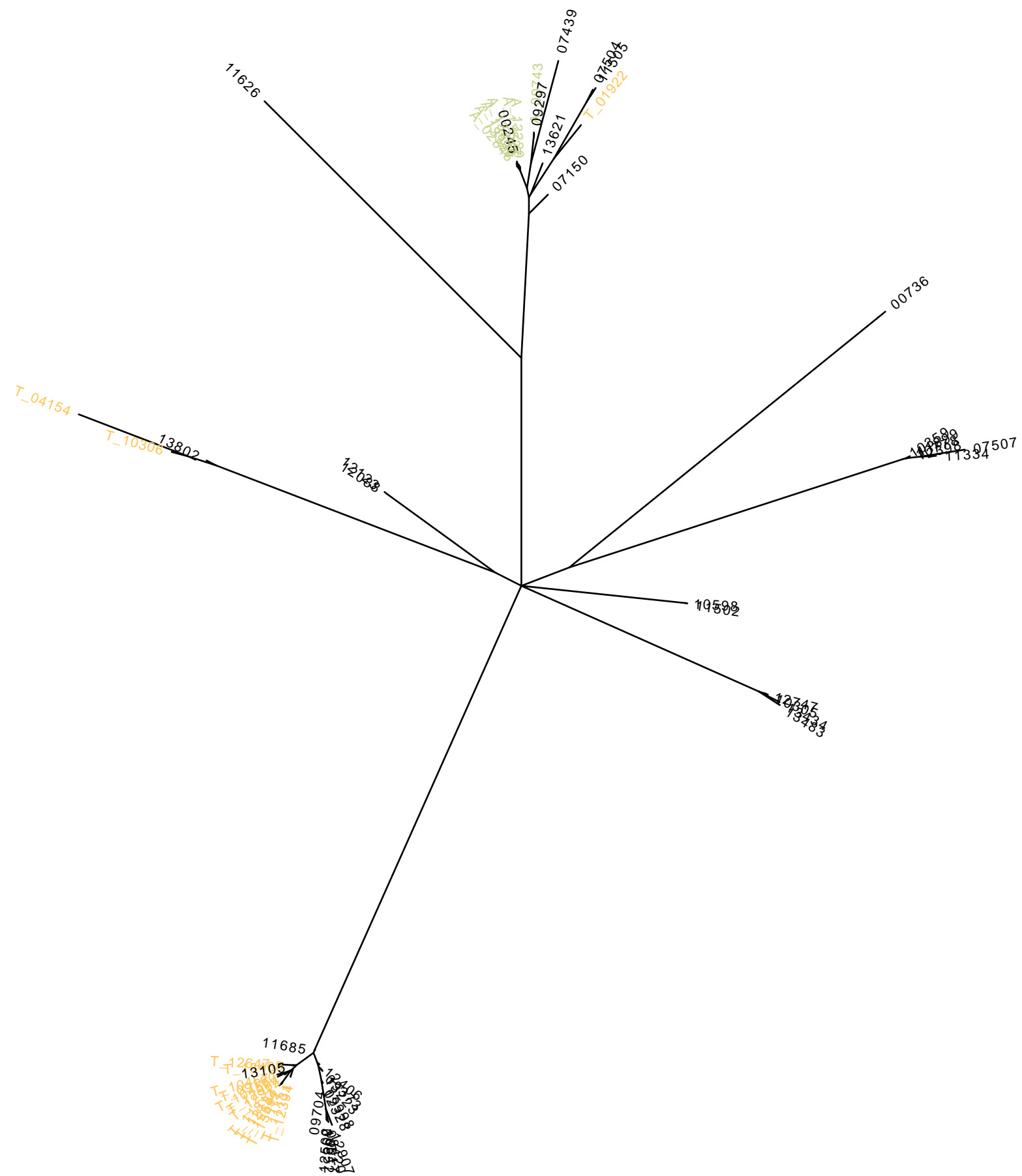

Supplement: Figure S3 — Neighbor-joining tree of the protein sequences of GP63 genes; numbers correspond to gene ID. Differentially up-regulated in A (green), differentially up-regulated in T (orange). [file peerj-05-3017-s012.pdf]

A

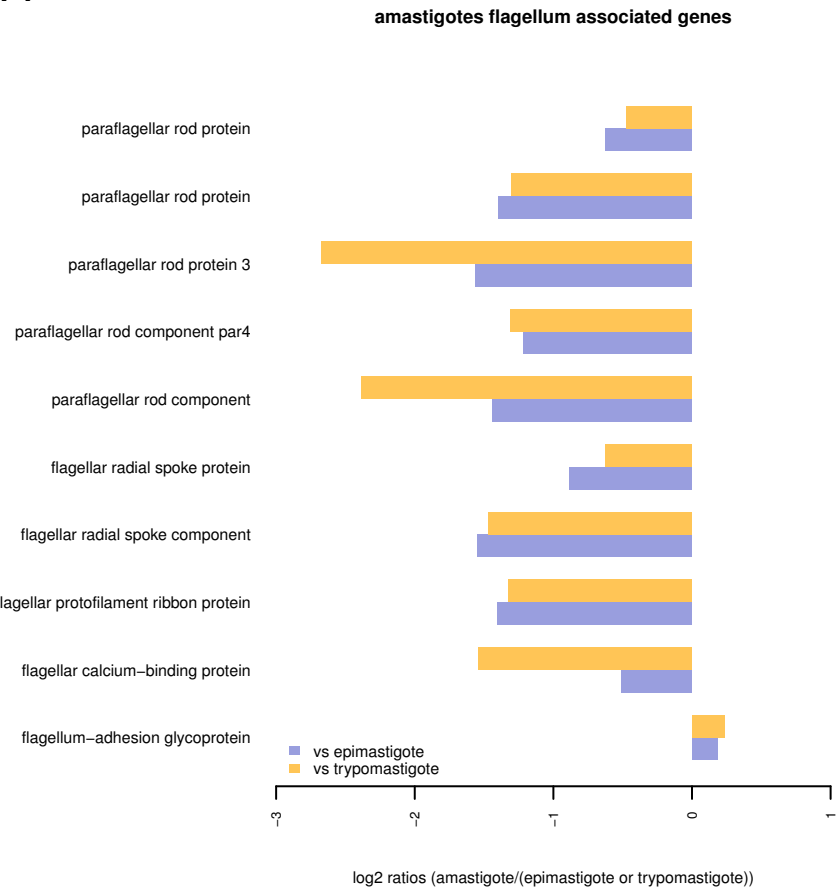

B

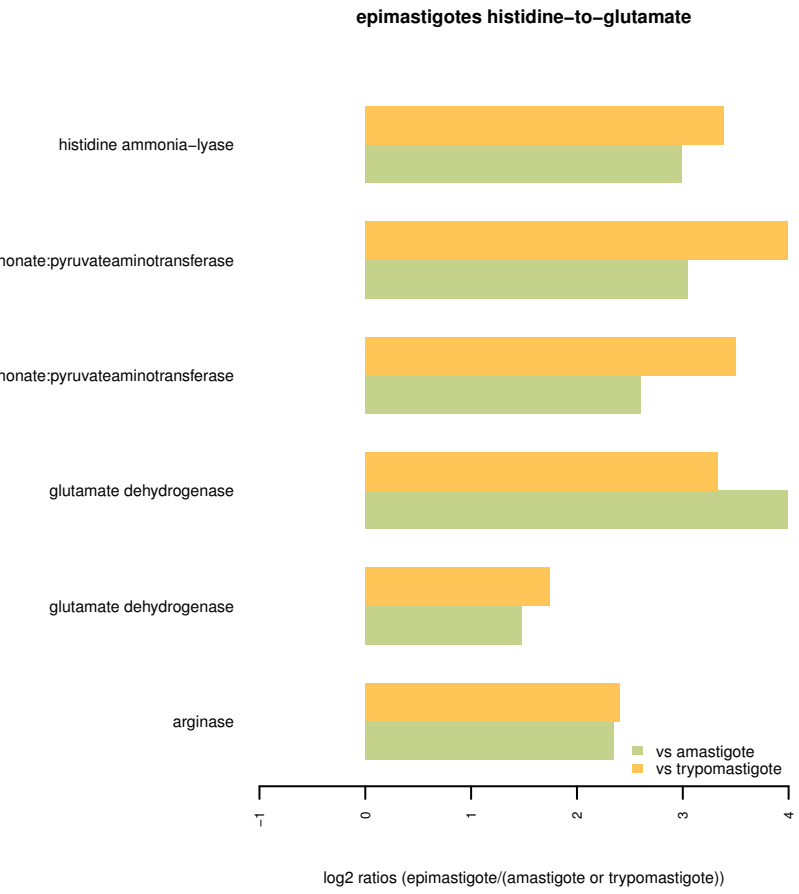

C

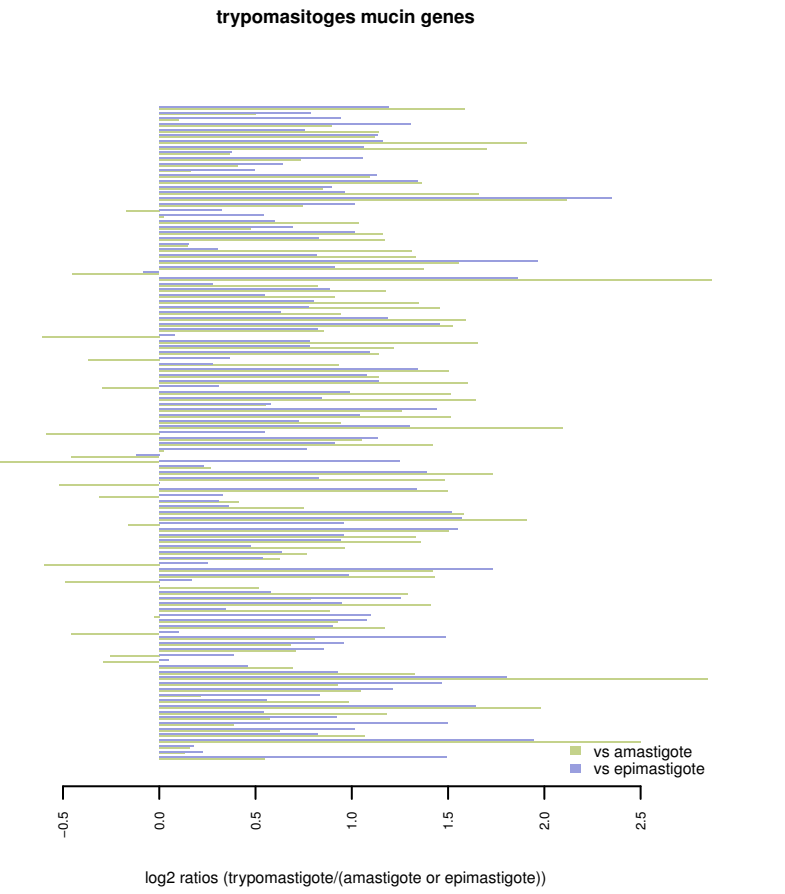

Supplement: Figure S4 — Comparison to known protein expression profiles. (A) Flagellum associated genes down-regulated in amastigotes. (B) Genes related to conversion of histidine to glutamate up-regulated in epimastigotes. (C) Mucins up regulated in trypomastigotes. [file peerj-05-3017-s013.pdf]
